# Agreement Between Reasoning-Oriented Generative AI Models and Clinical Educators in Evaluating Japanese Objective Structured Clinical Examination Transcripts: Preliminary Comparative Study

**DOI:** 10.2196/92016

**Published:** 2026-07-02

**Authors:** Takanobu Hirosawa, Masashi Yokose, Tetsu Sakamoto, Arisa Hayashi, Yukinori Harada, Kazuki Tokumasu, Kazuya Mizuta, Taro Shimizu

**Affiliations:** 1 Department of Diagnostic and Generalist Medicine, Dokkyo Medical University, 880 Kitakobayashi, Mibu-cho, Shimotsuga, Tochigi, 321-0293, Japan, 81 282-87-2498; 2 Department of General Medicine, Okayama University Graduate School of Medicine, Dentistry and Pharmaceutical Sciences, Okayama, Japan; 3 Department of Intensive Care Medicine, Kameda Medical Center, Kamogawa, Chiba, Japan

**Keywords:** artificial intelligence, generative artificial intelligence, medical interview training, standardized patient, simulation education

## Abstract

**Background:**

Medical interview training faces limitations in both implementation and evaluation. While generative artificial intelligence (GenAI) offers a potential solution, it remains unclear whether reasoning-oriented models improve evaluation, particularly for the Japanese language.

**Objective:**

We assessed scoring patterns to evaluate the agreement between reasoning-oriented GenAI model scores and clinical educator consensus ratings in Japanese medical interview training.

**Methods:**

This study was conducted at a medical university in Japan using original Japanese-language text data derived from medical interview training. Postgraduate year 1 and 2 residents were involved. Two blinded human clinical educators independently evaluated the transcripts and reached consensus through discussion. These consensus ratings were used as a practical reference standard, while preconsensus agreement was also assessed to characterize interhuman variability. Two GenAI models, GPT-5.2 Thinking (OpenAI) and Gemini 3.0 Pro (Google LLC), independently evaluated the same transcripts directly. Each GenAI model generated a single zero-shot evaluation per transcript using default settings. All evaluations used a standardized 6-domain Objective Structured Clinical Examination rubric (patient care, history taking, physical examination, accuracy and organization of clinical information, clinical reasoning, and management) scored on a 1‐6 Likert scale, where 1 indicates inferior, and 6 indicates excellent. We compared mean evaluation scores using the Wilcoxon signed-rank test and assessed interrater reliability using intraclass correlation coefficients between the GenAI models and the clinical educators.

**Results:**

Clinical educators and both GenAI models rated the entire dataset of 40 transcripts by 20 included residents. Clinical educator consensus ratings yielded the highest overall mean scores (5.18, 95% CI 5.06 to 5.30). Comparatively, both GenAI models demonstrated significantly lower scores: GPT-5.2 Thinking assigned the lowest overall score (3.68, 95% CI 3.62 to 3.72; *P*<.001), followed by Gemini 3.0 Pro (4.09, 95% CI 3.97 to 4.21; *P*<.001). This discrepancy was most pronounced in the management domain, where GPT-5.2 Thinking assigned 2.93 (95% CI 2.79 to 3.06) compared with the clinical educator consensus mean score of 5.20 (95% CI 4.91 to 5.49). Agreement between the GenAI models and the clinical educator consensus ratings was poor across all domains, with overall intraclass correlation coefficients of 0.04 (95% CI 0.00 to 0.09) for GPT-5.2 Thinking and 0.22 (95% CI 0.10 to 0.35) for Gemini 3.0 Pro.

**Conclusions:**

In this preliminary, single-center, transcript-based Japanese-language study, single-run zero-shot evaluations by GPT-5.2 Thinking and Gemini 3.0 Pro showed lower scores and poor agreement with the clinical educator consensus ratings. These findings should be interpreted cautiously because multiple outputs, prompt-sensitivity analyses, local validation, and model-parameter comparisons were not performed. Under the specific conditions tested, these models should not be used as standalone evaluators for Japanese Objective Structured Clinical Examination medical interview transcripts. Whether these models can provide useful formative feedback remains a hypothesis.

## Introduction

### Importance of Medical Interview Training

Medical interviewing is a cornerstone of modern health care, including medical education and practice [1-3]. Mastering clinical communication is not merely about gathering information; it is a critical bedside skill required to build rapport, accurately estimate pretest probability, and formulate differential diagnoses [4,5]. Accurate estimation of pretest probability is essential for guiding effective clinical investigations, thereby achieving diagnostic excellence [6-10]. Consequently, the ability to perform a structured and empathetic medical interview is a fundamental competency that medical learners must demonstrate before clinical practice [11].

### Challenges in Traditional Educational Methods

Despite its importance, effective training in medical interviewing faces several challenges in implementation [12,13]. Unlike knowledge-based disciplines, which can be relatively easily scaled through mass lectures, digital media, or textbooks, clinical skills training is inherently resource-intensive [14]. Traditional methods, such as Objective Structured Clinical Examinations (OSCEs), require substantial investments in time, funding, and personnel, specifically due to the burden of recruiting and training standardized patients and securing clinical educators for supervision [15-20]. In resource-limited environments or institutions facing faculty shortages, providing medical learners with sufficient opportunities for medical interview training is often unfeasible [21]. Consequently, there is a growing disparity between the need for extensive clinical skills training and the educational infrastructure available to support it.

Furthermore, achieving objective and consistent evaluation remains a persistent difficulty, even within structured frameworks such as the OSCE. Although the OSCE was designed to standardize assessment through checklists and rating scales, human evaluation is inevitably subject to interrater variability and cognitive biases [22,23]. Studies have shown that interrater reliability can fluctuate due to factors such as variability in examiner stringency, known as the “hawk-dove” effect [24], and the “halo” effect, where an examiner’s general impression of a student influences checklist scores [25]. In the context of medical education, human evaluators often struggle to provide immediate and actionable feedback due to time constraints, limiting the educational value of the assessment for the learner [26].

### Emergence of Artificial Intelligence in Medical Education

To address these systemic limitations, digital technology, including artificial intelligence (AI), has rapidly emerged as a transformative tool in health care, especially medical education [27-29]. Historically, rule-based AI applications were limited to rigid simulations or diagnostic algorithms [30]. However, recent advancements have expanded the utility of AI to include personalized learning assistants, virtual patient simulations, and automated feedback systems [31].

### Generative Artificial Intelligence and Natural Language Processing

Generative artificial intelligence (GenAI), a subfield of AI that uses natural language processing, holds promise for medical education [32,33]. These systems are powered by large language models (LLMs), which process and generate human-like text by predicting word sequences based on probabilistic models trained on massive datasets [34].

Unlike earlier rule-based AI, modern LLMs can parse complex, unstructured data—such as the dialogue of a medical interview—and generate contextually relevant responses or analyses [35,36]. Two of the most prominent state-of-the-art generative models currently available are generative pretrained transformer (GPT) and Gemini, developed by OpenAI and Google, respectively [37-39]. These general-purpose, unspecialized platforms have demonstrated remarkable capabilities in medical reasoning and performance on licensing examinations [40-42]. However, their utility is not limited to answering questions; they possess the potential to act as evaluators, assessing medical learners’ performance against established clinical criteria.

### Previous Work

In a preceding investigation, we explored the feasibility of using the previous GenAI model, GPT-4 (OpenAI), as a supplemental evaluation tool for Japanese OSCEs [43]. This experimental study compared GPT-4’s evaluation of medical students against the consensus of experienced physicians. The results highlighted a dichotomy in performance: while GPT-4 provided evaluation scores comparable to clinical educator consensus ratings in the domains of patient care or communication and history taking, the GenAI model overestimated student performance in physical examination, clinical reasoning, and patient notes compared to the physicians. Furthermore, the agreement between the GenAI and the clinical educator consensus ratings, as measured by intraclass correlation coefficients (ICCs), remained low across most domains. These findings suggested that while GenAI possesses the potential to supplement medical education, particularly in communication-based tasks, its reliability as a standalone evaluator remains unproven, and tendencies toward score inflation require attention.

### Reasoning-Oriented LLMs

Furthermore, the latest evolution in LLMs introduces reasoning capabilities, often referred to as chain-of-thought processing [44]. Unlike standard models that predict the next word based on probability, recent GenAI platforms include reasoning-oriented models designed to improve performance on tasks requiring multistep reasoning [45]. Although such systems may generate outputs consistent with more extensive reasoning processes, their internal reasoning mechanisms were not directly accessible or analyzed in the present study. The impact of reasoning-oriented model design on the subjective evaluation of medical trainees, therefore, remains uncertain.

### Gaps and Study Aim

While the capabilities of reasoning-oriented GenAI models are well-documented, their validity as evaluators of clinical skills remains underexplored, particularly in non-English contexts [46,47]. The nuances of the Japanese language—which relies heavily on context, honorifics, and indirect communication—pose unique challenges for LLMs compared to English [48]. Given the contextual complexity of the Japanese language, it is currently unknown whether general-purpose GenAI models such as GPT and Gemini, especially reasoning-oriented models, can accurately evaluate Japanese medical interviews with a level of reliability comparable to that of clinical educators.

Therefore, this preliminary study aims to assess the agreement between reasoning-oriented GenAI models and clinical educator consensus ratings in the evaluation of Japanese OSCEs. Specifically, we aim to compare the evaluation scores generated by these GenAI models against those provided by experienced clinical educators to determine if GenAI can serve as a reliable, resource-efficient adjunct in medical training. Demonstrating alignment with human experts would provide preliminary support for these tools as scalable adjuncts to address faculty limitations and standardize assessment.

## Methods

### Setting

This study was conducted as a preliminary comparative analysis within the Department of Diagnostic and Generalist Medicine (general internal medicine) at Dokkyo Medical University, Tochigi, Japan.

**Figure 1.**
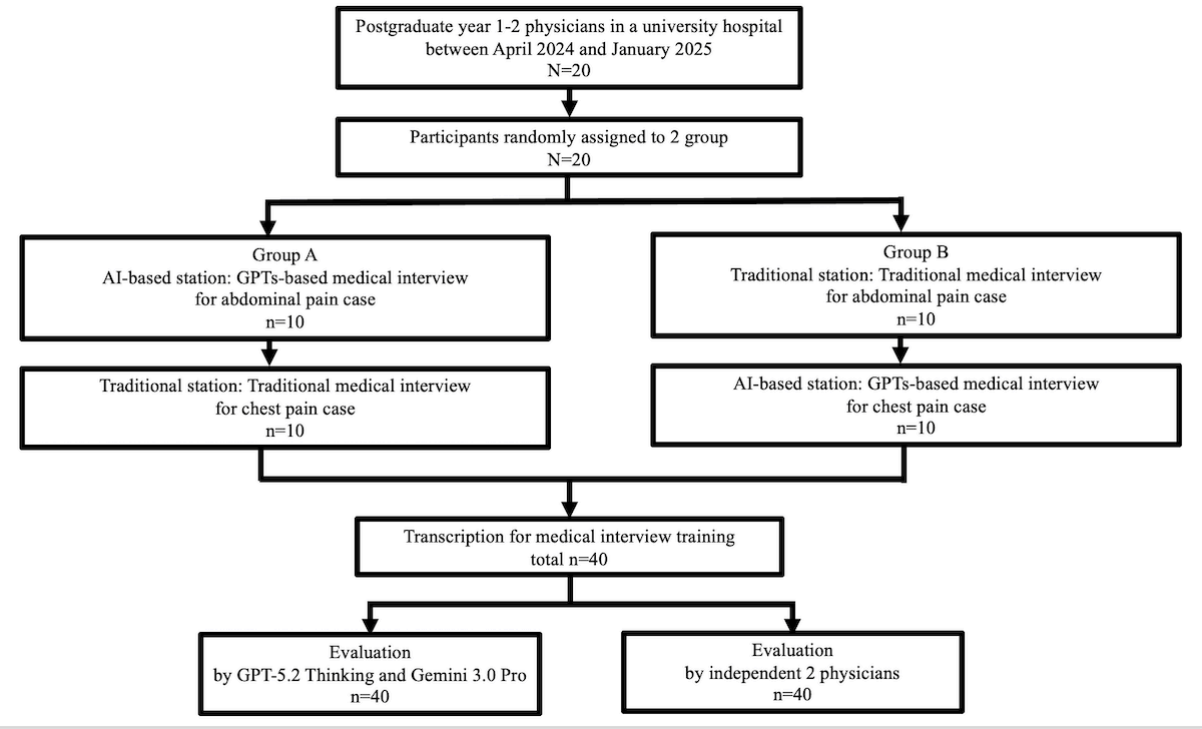
The flowchart includes participants, group allocation, and evaluation by medical educators, as well as both generative artificial intelligence models. GPT: generative pretrained transformer.

The current research used a dataset derived from our prior investigation into the utility of GenAI for Japanese medical interview training [49]. This study consisted of three phases. First, we prepared text data from medical interview training sessions. Second, human clinical educators evaluated these data. Third, GenAI models evaluated the same text data. The entire study, including all medical interview training, transcripts, and evaluations, was conducted in Japanese; no translated versions were used. Both GenAI models evaluated the original Japanese-language transcripts directly; no English translations or other translated versions were used for model evaluation. The flowchart, including participants, group allocation, and evaluation by clinical educators and both GenAI models, is shown in Figure 1.

The University Hospital Medical Information Network registration refers to the original medical interview training study from which the transcript dataset was derived. The present comparative evaluation of GenAI-generated and clinical educator scores was conducted as a secondary analysis of those previously collected transcripts and was not separately prospectively registered.

### Ethical Considerations

This study protocol was reviewed and approved by the Institutional Review Board of the Dokkyo Medical University Hospital (No. R-79‐14J). All participants provided written informed consent. No additional platform-level data retention settings were available or verified through the interfaces used at the time of evaluation. Only the anonymized text required for evaluation was submitted.

### Text Data From Medical Interview Training

We recruited postgraduate year 1 (PGY-1) and postgraduate year 2 (PGY-2) residents rotating through the general internal medicine department. Participants were randomized to a crossover sequence alternating between two distinct training styles to prepare this study’s data: (1) a text-based medical interview training modality using a custom GPT chatbot to simulate a patient, hereafter referred to as the chatbot-based training style, and (2) a traditional face-to-face medical interview training with a human standardized patient, referred to as the traditional training style. Subsequently, the text data derived from both training styles were anonymized and independently evaluated by three evaluator sources: the clinical educator consensus ratings, GPT-5.2 Thinking, and Gemini 3.0 Pro. The GPTs, developed by OpenAI, were custom GPT-based chatbots [50], configured via a noncoding interface to simulate specific patient personas.

Each participant completed 2 stations. The first station featured a case of abdominal pain, and the second station featured a case of chest pain. Both training styles, chatbot-based and traditional, were applied to these identical clinical cases. To ensure consistency, participants in both styles were only required to state or type the specific physical examination maneuvers they intended to perform. Following the medical interview part, the participants were required to present their clinical assessment and plan for each case. All training sessions were video-recorded to facilitate transcription. The detailed protocols for the medical interview training are provided in Multimedia Appendix 1. Summaries of the two clinical cases used in medical interview training are shown in Multimedia Appendix 2.

No formal sample size calculation was performed because this was a preliminary exploratory study using available transcripts from a medical interview training program. The sample size was determined by the number of participating residents and completed training transcripts during this study’s period.

### Data Extraction and Preparation

Following the medical interview training sessions, the content of all medical interviews and subsequent case presentations was extracted as text data for analysis. For the chatbot-based sessions, text data were generated by directly extracting the conversation logs. For the traditional sessions, the dialogue was manually transcribed by the primary researcher (TH) from the video recordings. To ensure the evaluations focused solely on content, the text data were formatted to remove identifiers of the modality used, including specific formatting unique to the chatbot interface. Although identifiers of the training modality and formatting elements unique to the chatbot interface were removed before evaluation, residual differences in transcript length, verbosity, interaction structure, completeness, or transcription characteristics could remain because chatbot-based records were extracted directly from conversation logs, whereas traditional sessions were manually transcribed from video recordings. As an exploratory analysis, we compared transcript length, measured as the total number of Japanese characters, between the two training modalities using the paired Wilcoxon signed-rank test. An example of anonymized transcription formatting is provided in Textbox 1.

Textbox 1. **An example of anonymized transcription formatting. The original transcription was written in Japanese.**

Physician: When did the pain start, and where exactly did it hurt?

Patient: It started two weeks ago. The pain is strongest in the pit of my stomach (epigastrium).

Physician: What kind of pain is it?

Patient: It feels like a burning pain.

(Continue medical interview training)

Physician:

The problem list includes epigastric pain and a positive Murphy’s sign.

Regarding the differential diagnosis, I first considered acute conditions such as cholecystitis, myocardial infarction, aortic dissection, and pulmonary thromboembolism.

(Continue physician’s presentation)

### Evaluation by Clinical Educators

Two independent clinical educators (MY and T Sakamoto) evaluated the anonymized text data. The evaluation criteria were based on standard OSCE scoring rubrics with a 6-point Likert scale, where 1 is inferior and 6 is excellent [51,52]. The assessment covered six specific domains: (1) patient care and communication skills, (2) thoroughness of history-taking, (3) physical examination proficiency, (4) accuracy and organization of clinical information (evaluated for the logical flow and organization of the clinical facts presented, rather than formal medical recording standards), (5) clinical reasoning capability, and (6) overall patient management strategies. Crucially, both evaluators were blinded to the training modality, chatbot-based vs traditional, corresponding to each transcript. The clinical educators evaluated the same text-only anonymized transcripts that were provided to the GenAI models and did not have access to video recordings, audio recordings, training modality information, participant identities, or any additional contextual information from the training sessions. The two clinical educators first completed their evaluations independently and without access to one another’s scores. Their original independent ratings were preserved before consensus discussion and were used for the preconsensus interrater agreement analysis. For transcripts with discrepant scores, the educators reviewed the six rubric domains and discussed the relevant transcript content with reference to the predefined rubric descriptors. A consensus score was then assigned for each domain. No third adjudicator was used because all discrepancies were resolved through discussion between the two educators. To control potential order effects, the sequence of evaluations was randomized using a randomization list generated via Microsoft Excel by another researcher (KM). The detailed evaluation rubric for medical interview training is presented in Multimedia Appendix 3.

### Evaluation by GenAI

#### Overview

For GenAI evaluation, we selected two state-of-the-art reasoning-oriented GenAI models: GPT-5.2 Thinking, developed by OpenAI, and Gemini 3.0 Pro, developed by Google LLC. These models were selected for their advanced reasoning-oriented capabilities and accessibility.

Both models were tasked with evaluating the entire dataset of interview and presentation transcripts. The same anonymized text data evaluated by the clinical educators was fed into both GenAI models. Each evaluation was based on a single generated output per transcript by each model. Repeated evaluations, alternative prompts, and parameter-tuning experiments were not performed in the primary analysis. No worked scoring examples, educator-approved anchor cases, few-shot examples, or model-specific calibration materials were supplied to either GenAI model. To prevent context retention between cases, the model context was refreshed after every individual evaluation session. The specific prompt engineering used to guide the GenAI models’ evaluations was standardized as follows in Textbox 2. The GenAI models were instructed to provide numerical scores for each rubric domain. Qualitative feedback or narrative comments were not requested, collected, or analyzed in this study. GPT-5.2 Thinking was accessed through the ChatGPT (OpenAI) web interface. Gemini 3.0 Pro was accessed via Google AI Studio. All GenAI-based evaluations were executed on January 9, 2026.

Available settings were kept at the platform defaults. For GPT-5.2 Thinking, model parameters such as temperature, top-p, and output length were not visible or adjustable in the ChatGPT web interface used in this study; therefore, the platform default settings were used. For Gemini 3.0 Pro, the following Google AI Studio settings were used: temperature, 1.0 on a scale from 0 to 2; thinking level, high, selected from low, medium, and high; top-p, 0.95 on a scale from 0 to 1; and output length, 65,536 tokens. No additional user-defined system-level instructions were used beyond the standardized evaluation prompt.

Textbox 2. **Specific prompt engineering used to guide the generative artificial intelligence models’ evaluations.**

You are a professional evaluator in the following medical interview training. Please evaluate the medical interview, assessment, and plan below using the A–F criteria, assigning a score on a 1–6 scale.

A. Patient care/ communication

6. Can perform independently (can be trusted to perform independently)

5. Can perform without direct supervision by a supervising physician

4. Can perform under direct supervision by a supervising physician

3. There is a risk of being unable to establish an appropriate physician–patient relationship

2. Unable to establish an appropriate physician–patient relationship

1. Causes significant harm to the patient

B. Medical interview

6. Can perform independently (can be trusted to perform independently)

5. Can perform without direct supervision by a supervising physician

4. Can perform under direct supervision by a supervising physician

3. Insufficient information gathering, with potential interference with clinical care

2. Insufficient information gathering, interfering with clinical care

1. Almost no information gathered, clearly interfering with clinical care

C. Physical examination

6. Can perform independently (can be trusted to perform independently)

5. Can perform without direct supervision by a supervising physician

4. Can perform under direct supervision by a supervising physician

3. Potential to interfere with clinical care

2. Interferes with clinical care

1. Clearly interferes with clinical care

D. Accuracy and organization of clinical information

6. Information obtained from the interview is organized logically and presented accurately, without excess or omission.

5. Information is accurate without excess or omission, but lacks systematic organization.

4. Most information obtained from the interview is included and clear.

3. Insufficient organization of information, with potential interference with clinical care.

2. Information is disorganized or insufficient, interfering with clinical care.

1. Almost no coherent information is presented; clearly interfering with clinical care.

E. Clinical reasoning

6. Lists differential diagnoses comprehensively and explains them rationally

5. Lists differential diagnoses and explains them rationally

4. Can rationally explain only the primary diagnosis

3. Lists differential diagnoses only superficially

2. Unable to list appropriate differential diagnoses

1. Unable to list differential diagnoses

F. Management

6. Fully formulates appropriate and specific plans with proper prioritization to diagnose differential diagnoses

5. Formulates a small number of appropriate and specific plans with proper prioritization to diagnose differential diagnoses

4. Formulates appropriate and specific plans to diagnose differential diagnoses, but without appropriate prioritization

3. Formulates appropriate plans to diagnose differential diagnoses, but lacks specificity

2. Formulates plans to diagnose differential diagnoses, but they are inappropriate

1. Unable to formulate plans to diagnose differential diagnoses

Note: For the physical examination, assume that the examination was performed for the physical findings entered as “desired physical findings” in the medical interview training below.*******************************************************************

(copy and paste text data extracted by medical interview training)

*******************************************************************

#### Data Collection and Outcomes

We collected background data, including participants’ biological sex and their years since obtaining a medical degree. Additionally, the length of each medical interview transcript was recorded as the total number of Japanese characters in the original Japanese transcript.

The primary outcome was the difference in the overall OSCE evaluation score among the three evaluator groups: clinical educator consensus score, GPT-5.2 Thinking, and Gemini 3.0 Pro. The overall score was defined as the mean score across the six rubric domains. Pairwise comparisons of the overall score were performed among the three evaluator groups. The secondary outcomes included domain-level score differences across the six rubric domains and interrater agreement between each GenAI model and the clinical educator consensus score, assessed using ICCs. Domain-level score comparisons were considered exploratory.

Additionally, as an exploratory outcome, we assessed the intermodel reliability (agreement between GPT-5.2 Thinking and Gemini 3.0 Pro). Furthermore, we investigated the influence of interaction modality by comparing overall scores and agreement specifically between the chatbot-based and the traditional styles.

### Statistical Analysis

Evaluation scores are presented as means with 95% CIs. Differences in scores between groups (clinical educator consensus score vs GPT-5.2 Thinking and clinical educator consensus score vs Gemini 3.0 Pro) were analyzed using the Wilcoxon signed-rank test. To better reflect the ordinal nature of the rubric, medians and IQRs are also reported in Multimedia Appendix 4.

To characterize the reference standard, we assessed preconsensus agreement between the two clinical educators using ICCs based on a two-way random-effects model for absolute agreement and single measurements. We also calculated exact agreement, agreement within 1 point, mean absolute difference, maximum absolute difference, and mean difference. These metrics were calculated by domain and for the overall score.

Agreement between evaluators was assessed using ICCs calculated with a two-way random-effects model for absolute agreement and single measurements. This model treats both participants and raters as random effects and evaluates whether individual ratings from each GenAI model agree with those of the human clinical educators. ICC estimates are reported together with their corresponding 95% CIs. The degree of reliability was interpreted according to the following guidelines: ICC values less than 0.5 indicate poor reliability; values between 0.5 and 0.75 indicate moderate reliability; values between 0.75 and 0.9 indicate good reliability; and values greater than 0.9 indicate excellent reliability [53].

As each resident completed two stations, the 40 transcript-level observations were not fully independent. To address this repeated structure, we conducted a participant-level sensitivity analysis for the primary outcome. For each resident, the overall scores from the two stations were averaged within each evaluator group, yielding one person-level overall score per resident for clinical educators, GPT-5.2 Thinking, and Gemini 3.0 Pro. We then repeated the paired comparisons using Wilcoxon signed-rank tests at the participant level (n=20).

We did not fit a mixed-effects model in the present preliminary analysis because the dataset included only 20 residents and two stations representing two clinical cases. With only two case levels and a modest sample size, simultaneous estimation of participant-level and case-level random effects would have limited stability and interpretability. Future studies with larger numbers of participants and stations should use mixed-effects models to account for resident-level clustering and case effects.

Given the multiple comparisons performed in this study, we applied the Bonferroni correction to adjust the significance levels and control the family-wise error rate [54]. For the primary confirmatory analysis, Bonferroni correction was applied to the three pairwise comparisons of the overall OSCE score among the evaluator groups: GPT-5.2 Thinking vs clinical educator consensus score, Gemini 3.0 Pro vs clinical educator consensus score, and GPT-5.2 Thinking vs Gemini 3.0 Pro. Accordingly, the Bonferroni-corrected significance threshold for the primary analysis was *P*<.017 (.05/3). Domain-level score comparisons across the six rubric domains were conducted as secondary exploratory analyses and are reported with exact *P* values; these analyses were not included in the primary Bonferroni correction. ICC analyses were interpreted descriptively using established reliability thresholds and were not subjected to Bonferroni correction. All statistical analyses were performed using R software (version 4.2.2; R Foundation for Statistical Computing).

## Results

### Characteristics

A total of 20 physicians participated in this study. Among participants, 55% (11/20) were PGY-1 and 45% (9/20) were PGY-2. Further, 90% (18/20) of participants were male, indicating a predominantly male sample. This sex imbalance should be considered when interpreting the generalizability of the findings to more sex-balanced resident cohorts.

Regarding text data, a total of 40 medical interview transcripts were analyzed, including 20 chatbot-based transcripts and 20 traditional-style transcripts. Clinical educators and both GenAI models rated the entire dataset of 40 transcripts. Japanese character count ranged from 872 to 5167 (mean 1769, 95% CI 1463 to 2074).

### Evaluation Scores: Comparison Between Reasoning-Oriented GenAI Models and Clinical Educators

Table 1 summarizes the comparison of scores assigned by GPT-5.2 Thinking, Gemini 3.0 Pro, and the clinical educator consensus ratings. Across all domains, the clinical educator consensus ratings were higher than the GenAI model scores.

For the primary outcome, the clinical educator consensus mean score was 5.18 (95% CI 5.06 to 5.30). In contrast, Gemini 3.0 Pro assigned a significantly lower mean score of 4.09 (95% CI 3.97 to 4.21; *P*<.001), and GPT-5.2 Thinking assigned the lowest score of 3.68 (95% CI 3.62 to 3.72; *P*<.001). The median overall score was 5 (IQR 5‐6) for the clinical educator consensus rating, 4 (IQR 3‐4) for GPT-5.2 Thinking, and 4 (IQR 3‐5) for Gemini 3.0 Pro. Median and IQR values for each evaluator group and domain are detailed in Multimedia Appendix 4.

**Table 1.** Comparison of evaluation scores between the two GenAI^a^ models, GPT-5.2 Thinking and Gemini 3.0 Pro, and the clinical educator consensus score.

	Scores with a 6-point Likert scale, mean (95% CI)			*P* value^b^		
	GPT-5.2 Thinking	Gemini 3.0 Pro	Clinical educator consensus score	GPT-5.2 Thinking vs clinical educator consensus score	Gemini 3.0 Pro vs clinical educator consensus score	GPT-5.2 Thinking vs Gemini 3.0 Pro
Overall	3.68 (3.62‐3.72)	4.09 (3.97‐4.21)	5.18 (5.06‐5.30)	<.001	<.001	<.001
Patient care and communication	4.08 (3.99‐4.16)	4.43 (4.17‐4.68)	5.25 (5.00‐5.50)	<.001	<.001	.014
History taking	3.83 (3.68‐3.97)	4.38 (4.14‐4.60)	5.10 (4.90‐5.30)	<.001	<.001	<.001
Physical examination	3.90 (3.74‐4.06)	4.08 (3.70‐4.45)	5.45 (5.22‐5.68)	<.001	<.001	.38
Accuracy and organization of clinical information	3.83 (3.70‐3.95)	4.05 (3.75‐4.34)	5.05 (4.82‐5.28)	<.001	<.001	.14
Clinical reasoning	3.50 (3.28‐3.72)	4.05 (3.69‐4.41)	5.03 (4.71‐5.34)	<.001	<.001	<.001
Management	2.93 (2.79‐3.06)	3.58 (3.28‐3.87)	5.20 (4.91‐5.49)	<.001	<.001	<.001

a GenAI: generative artificial intelligence.

b Statistical comparisons were conducted using the Wilcoxon signed-rank test. Bonferroni correction was applied to the 3 primary pairwise comparisons of the overall OSCE score, with a corrected significance threshold of *P*<.017. Domain-level comparisons were secondary exploratory analyses and were not included in the primary Bonferroni correction.

This trend of lower GenAI scores persisted across every subdomain. The discrepancy was most pronounced in the management domain, where the clinical educator consensus mean score was 5.20 (95% CI 4.91 to 5.49), whereas GPT-5.2 Thinking gave a score of 2.93 (95% CI 2.79 to 3.06), and Gemini 3.0 Pro gave 3.58 (95% CI 3.28 to 3.87). Similarly, in physical examination, human evaluators gave high marks (mean 5.45, 95% CI 5.22 to 5.68), while both GPT-5.2 Thinking (mean 3.90, 95% CI 3.74 to 4.06) and Gemini 3.0 Pro (mean 4.08, 95% CI 3.70 to 4.45) rated the performance significantly lower.

As each resident contributed two transcripts, we conducted a participant-level sensitivity analysis using the mean overall score across the two stations for each resident. The findings were consistent with the transcript-level analysis. At the participant level, clinical educator consensus ratings yielded the highest mean overall score (5.18, 95% CI 4.99 to 5.37), followed by Gemini 3.0 Pro (4.09, 95% CI 3.91 to 4.28) and GPT-5.2 Thinking (3.68, 95% CI 3.59 to 3.76). Paired participant-level comparisons showed that GPT-5.2 Thinking scored lower than the clinical educator consensus score (mean difference −1.50, 95% CI −1.68 to −1.32; *P*<.001), Gemini 3.0 Pro scored lower than the clinical educator consensus score (mean difference −1.09, 95% CI −1.26 to −0.91; *P*<.001), and Gemini 3.0 Pro scored higher than GPT-5.2 Thinking (mean difference 0.42, 95% CI 0.25 to 0.58; *P*<.001). These results were consistent with the primary transcript-level analysis after participant-level aggregation. Participant-level sensitivity analysis of overall scores is provided in Multimedia Appendix 5.

### Preconsensus Agreement Between Human Clinical Educators

As shown in Table 2, agreement between the two clinical educators was poor. For the overall score, the preconsensus ICC was 0.14 (95% CI 0.05 to 0.23). Exact agreement for the overall score was 0.0%, and agreement within 1 point was 47.5%. The mean absolute difference in overall score was 1.13 points, with a maximum absolute difference of 2 points. The mean signed difference was 1.12 points, indicating that one educator tended to assign higher overall scores than the other educator before the consensus discussion.

These findings indicate that substantial interhuman variability existed before consensus-building. Therefore, the consensus score was used as a practical reference standard, but poor GenAI-human agreement should be interpreted in the context of preconsensus human rater variability and possible rubric subjectivity.

**Table 2.** Interrater agreement between the two human clinical educators.

	Interrater agreement (95% CI)	Exact agreement % (within 1 point %)	Mean absolute difference (maximum absolute difference)
Overall	0.14 (0.05‐0.23)	0 (47.5)	1.13 (2)
Patient care and communication	0.13 (0.00‐0.26)	5 (57.5)	1.38 (2)
History taking	0.13 (0.00‐0.28)	25 (80)	0.95 (2)
Physical examination	0.17 (0.01‐0.36)	5 (50)	1.45 (2)
Accuracy and organization of clinical information	0.05 (0.00‐0.14)	20 (65)	1.15 (2)
Clinical reasoning	0.26 (0.10‐0.41)	17.5 (70)	1.18 (3)
Management	0.30 (0.12‐0.47)	20 (75)	1.05 (2)

### Interrater Agreement

As shown in Table 3, the agreement between the GenAI models and the clinical educator consensus ratings was generally poor across all domains. The ICCs between GPT-5.2 Thinking and clinical educator consensus score were extremely low, ranging from 0.02 in management to 0.11 in accuracy and organization of information, with an overall agreement of 0.04 (95% CI 0.00 to 0.09), indicating poor reliability.

The agreement between Gemini 3.0 Pro and the clinical educator consensus score was slightly higher but still classified as poor. The overall ICC between Gemini 3.0 Pro and clinical educator consensus score was 0.22 (95% CI 0.10 to 0.35). The highest agreement for Gemini was observed in history taking (ICC 0.28) and the lowest in management (ICC 0.14).

As an additional post hoc exploratory analysis, we examined calibration-related characteristics of the GenAI model scores relative to the clinical educator consensus ratings. The mean signed difference was −1.50 points for GPT-5.2 Thinking and −1.09 points for Gemini 3.0 Pro. For the overall score, GPT-5.2 Thinking showed a Spearman rank correlation of 0.31 and an ordering concordance of 48.9%, whereas Gemini 3.0 Pro showed a Spearman rank correlation of 0.62 and an ordering concordance of 69.2%. These findings suggest that the disagreement was not explained solely by a constant additive score offset. A post hoc exploratory calibration analysis and three retrospective illustrative examples are provided in Multimedia Appendix 6.

**Table 3.** Interrater agreement between the two GenAI^a^ models, GPT-5.2 Thinking and Gemini 3.0 Pro, and the clinical educator consensus score.

	Interrater agreement (95% CI)		
	GPT-5.2 Thinking vs clinical educator consensus score	Gemini 3.0 Pro vs clinical educator consensus score	GPT-5.2 Thinking vs Gemini 3.0 Pro
Overall	0.04 (0.00‐0.09)	0.22 (0.10‐0.35)	0.28 (0.10‐0.43)
Patient care and communication	0.08 (0.01‐0.18)	0.24 (0.11‐0.39)	0.32 (0.15‐0.48)
History taking	0.09 (0.02‐0.19)	0.28 (0.14‐0.42)	0.35 (0.18‐0.50)
Physical examination	0.06 (0.00‐0.15)	0.18 (0.05‐0.32)	0.20 (0.05‐0.36)
Accuracy and organization of clinical information	0.11 (0.03‐0.21)	0.21 (0.08‐0.36)	0.26 (0.10‐0.42)
Clinical reasoning	0.05 (0.00‐0.13)	0.19 (0.06‐0.34)	0.31 (0.14‐0.46)
Management	0.02 (0.00‐0.08)	0.14 (0.03‐0.28)	0.25 (0.09‐0.40)

a GenAI: generative artificial intelligence.

### Exploratory Analysis

When comparing the two reasoning-oriented GenAI models from Table 1, Gemini 3.0 Pro generally assigned higher scores than GPT-5.2 Thinking. These differences were statistically significant in the domains of patient care (*P*=.01), history taking (*P*<.001), clinical reasoning (*P*<.001), and management (*P*<.001). However, there was no significant difference between the two GenAI models in the scores for physical examination (*P*=.38) and accuracy and organization of clinical information (*P*=.14).

As shown in Table 3, agreement between the two GenAI models (GPT-5.2 Thinking vs Gemini 3.0 Pro) was also poor, with an overall ICC of 0.28 (95% CI 0.10 to 0.43). The highest agreement between the models was found in history taking (ICC 0.35) and clinical reasoning (ICC 0.31).

Table 4 presents the evaluation scores classified by training style, chatbot-based vs traditional. For GPT-5.2 Thinking, no statistically significant difference in overall scores was detected between the chatbot-based and traditional styles (*P*=.44). For Gemini 3.0 Pro and clinical educator consensus score, mean scores were higher in the traditional style, but these differences did not reach statistical significance after Bonferroni correction (*P*=.03 for both; corrected threshold *P*<.017). Therefore, no statistically significant modality-related difference in overall scores was detected after correction. However, because this exploratory comparison involved a modest sample size and was not designed as an equivalence or noninferiority analysis, these findings should not be interpreted as evidence that assessment performance was robust or equivalent across training modalities.

Transcript length differed between the two training modalities. The median Japanese character count was 1754.5 (IQR 1395.3‐2558.3) for the traditional style and 1280.5 (IQR 1141‐1527) for the chatbot-based style. Traditional-style transcripts were significantly longer than chatbot-based transcripts (*P*=.001). Additional exploratory analyses of the associations between Japanese character count, evaluator scores, and absolute GenAI–clinical educator consensus score discrepancies are provided in Multimedia Appendix 7.

**Table 4.** Comparison of overall evaluation scores between the two GenAI^a^ models, GPT-5.2 Thinking and Gemini 3.0 Pro, and clinical educator consensus score according to training modality: chatbot-based and traditional styles.

Training modality	Scores with a 6-point Likert scale, mean (95% CI)		
	GPT-5.2 Thinking	Gemini 3.0 Pro	Clinical educator consensus score
Chatbot-based style (n=20)	3.63 (3.49‐3.77)	3.83 (3.56‐4.09)	4.89 (4.62‐5.16)
Traditional style (n=20)	3.72 (3.65‐3.79)	4.36 (4.14‐4.58)	5.47 (5.29‐5.64)
*P* value^b^	.44	.03	.03

a GenAI: generative artificial intelligence.

b Statistical comparisons were conducted using the Wilcoxon signed-rank test.

## Discussion

### Principal Results

This preliminary study evaluated the agreement between two reasoning-oriented GenAI models (GPT-5.2 Thinking and Gemini 3.0 Pro) and clinical educator consensus ratings when assessing Japanese medical interview training. The investigation yielded four critical findings regarding the comparative behavior of GenAI models vs human evaluation.

First, we observed systematically lower score assignment and poor relative agreement in both reasoning-oriented GenAI models. Clinical educator consensus ratings were generally high, mean scores exceeding 5 on a 6-point scale, reflecting a supportive, competency-based approach. This divergence was most profound in the management domain, where the mean score gap reached nearly 2.3 points between GPT-5.2 Thinking (mean 2.93) and clinical educator consensus score (mean 5.20). One possible explanation is that the models interpreted the rubric more literally or applied different scoring thresholds than the clinical educators. For example, clinical educators may have applied a pragmatic supervised-practice standard appropriate for PGY-1 and PGY-2 residents, whereas the models may have required more explicit documentation of comprehensive performance. However, this interpretation remains a hypothesis because model rationales were not collected, and item-level qualitative error analysis was not performed.

Second, this study highlights differences between the reasoning-oriented GenAI models. Gemini 3.0 Pro assigned higher scores than GPT-5.2 Thinking under the tested conditions. Specifically, Gemini 3.0 Pro consistently assigned significantly higher scores than GPT-5.2 Thinking across complex domains such as clinical reasoning and history taking. This observed difference suggests that the two platforms applied different scoring thresholds under the tested conditions. This study cannot determine whether the difference arose from model architecture, training data, alignment strategies, platform settings, or other factors.

Third, the reliability of these models for human assessment remains poor. The ICCs between each GenAI model and clinical educator consensus score were consistently below 0.5 across all domains, with agreement for GPT-5.2 Thinking being negligible (ICC=0.04). The low ICC between each GenAI and clinical educator consensus score indicates that the models ranked resident performance differently from the educator consensus ratings under the tested conditions. Consequently, the current unspecialized, untrained reasoning-oriented GenAI models should not be used as standalone alternatives under the tested conditions.

Importantly, the clinical educator consensus ratings were not an error-free gold standard. The two educators showed poor preconsensus agreement, indicating substantial interhuman variability and possible subjectivity in rubric application. Therefore, poor GenAI–human agreement may reflect both differences in model scoring behavior and instability in the human reference standard. Future validation studies should include larger educator panels, structured calibration exercises, and predefined anchor examples.

A retrospective review of three illustrative cases provided additional context for this hypothesis. In two transcripts with high clinical educator consensus ratings, the summarized assessment and plan were concise, but the clinical educators judged that the participants had appropriately addressed potentially life-threatening conditions and demonstrated performance suitable for supervised practice. The GenAI models assigned lower scores, particularly for clinical reasoning and management. In another relatively brief transcript, both the clinical educators and GenAI models assigned comparatively lower scores. These examples suggest that the degree of explicit documentation may influence scoring differently across evaluators. However, because the examples were selected retrospectively and model rationales were not collected, this interpretation remains hypothesis-generating.

Fourth, the exploratory analysis examined whether overall evaluation scores differed by training modality. No statistically significant difference was detected after correction between the chatbot-based and traditional training styles for any evaluator group. However, this finding should be interpreted cautiously because the subgroup comparison was exploratory and may have been underpowered. A lack of statistically significant difference does not establish equivalence or robustness across modalities. Larger studies specifically designed to evaluate modality effects are needed before drawing firm conclusions about whether transcript-based assessment is unaffected by training modality.

### Possible Explanations for Disagreement Between GenAI Models and Clinical Educators

Several mechanisms may explain the poor agreement between the GenAI models and the clinical educator consensus ratings. First, the GenAI models may have interpreted the rubric more literally than clinical educators. In actual OSCE evaluation, clinical educators may apply holistic judgment that considers the learner’s postgraduate level, overall safety, clinical plausibility, and adequacy for supervised practice. In contrast, the models may have treated each rubric descriptor as requiring comprehensive or textbook-level performance, thereby assigning lower scores when transcripts lacked explicit details.

Second, we did not perform a formal item-level qualitative error analysis of individual cases or model-generated rationales. Future studies should examine cases with large human-GenAI score discrepancies, classify the sources of disagreement, and determine whether calibration examples, few-shot prompting, or human-labeled training data can reduce systematic bias and improve alignment with clinical educator consensus ratings.

### Strengths of this Study

This study has several strengths. First, the inclusion of two reasoning-oriented GenAI platforms allows for a comparative assessment of platform-specific scoring behavior, showing that Gemini 3.0 Pro assigned higher scores than GPT-5.2 Thinking under the tested conditions. Second, we directly compared the evaluation performance of these reasoning-oriented models within a non-English context. The use of Japanese text data adds value by examining model evaluation behavior in a non-English, high-context linguistic setting, although language-specific mechanisms were not directly analyzed. Finally, the same anonymized text data evaluated by the clinical educators were fed into both reasoning-oriented GenAI models. This was complemented by a blind evaluation process for the clinical educators, minimizing potential bias.

### Limitations

Several limitations should be considered when interpreting these findings. First, this study was conducted using transcripts exclusively in the Japanese language and within a single institutional training context characterized by a predominantly male cohort (18/20, 90%). This demographic imbalance, combined with the single-center design, may limit generalizability to other languages, educational systems, and clinical environments. Although this study focused on Japanese-language transcripts, we did not directly analyze how Japanese-specific linguistic features influenced model scoring. Future studies should compare model performance across Japanese originals, English translations, and bilingual human ratings, and should examine whether specific Japanese communication features contribute to scoring disagreement.

Second, a central methodological limitation is that this study evaluated a single operational setup: one output per transcript, generated through zero-shot prompting without educator-approved calibration examples or rubric anchors. Repeated evaluations, prompt-sensitivity analyses, few-shot calibration, and systematic parameter comparisons were not performed [44,55]. Accordingly, the results do not establish the intrinsic capability of either model across alternative configurations. Specifically, only two reasoning-oriented GenAI models were evaluated at a single time point, and model performance may change substantially with future updates, parameter tuning, or alternative prompt engineering strategies. Consequently, the performance of other LLMs, such as Claude, developed by Anthropic, Llama, developed by Meta, and DeepSeek, by Hangzhou DeepSeek Artificial Intelligence Co, Ltd, remains unknown [56]. Within the OpenAI ecosystem, we did not evaluate the GPT-5.2 Pro model or ChatGPT Health (OpenAI) [57,58]. We used zero-shot prompting to ensure methodological consistency with the human evaluators. As LLM outputs may vary across multiple outputs, prompts, parameter settings, and model versions, this study could not estimate intramodel consistency or prompt sensitivity. Repeated evaluations using the same model versions would have strengthened the reliability assessment; however, the exact model versions used in the original evaluation were no longer accessible after subsequent platform updates. This limitation directly affects the interpretation of the reliability findings, as poor agreement with human educators may reflect not only systematic differences in scoring thresholds but also run-to-run variability. To address this, future research should incorporate one-shot or few-shot learning techniques [59,60].

Additionally, although standardized prompts were used and model context was reset between evaluations, stochastic variability inherent to LLMs may still influence scoring reproducibility. As only one output was generated per transcript, we could not quantify run-to-run variability [61]. Furthermore, the practical reference standard relied on consensus ratings from two clinical educators rather than a larger panel, which may limit the precision of the reference standard and restrict estimation of interhuman variability [62]. Although explicit modality identifiers and chatbot-specific formatting were removed, residual differences in transcript length, verbosity, dialogue structure, completeness, or transcription characteristics may have influenced the evaluation scores. Moreover, the evaluation was based solely on textual transcripts, excluding real physical examination, nonverbal communication cues, tone, and real-time interaction dynamics that are integral to authentic clinical assessment and may differentially influence human and GenAI judgment. In addition, the sample size was modest and included only two clinical scenarios. Therefore, this study may have limited statistical power for domain-specific analyses, including management-domain findings. The results may also be influenced by case specificity and may not generalize to other OSCE cases, learner levels, institutions, or clinical contexts. Although the participant-level sensitivity analysis addressed clustering by resident, this study still included only two clinical cases, and case-specific effects could not be fully separated from evaluator effects. Future studies with larger numbers of stations should use mixed-effects models to account simultaneously for participant, case, and evaluator-level variability. Finally, the reproducibility and governance of commercial GenAI-platform use depend partly on interface-level settings and platform policies, some of which may not be fully visible or independently verifiable to researchers.

Transcript length may also have influenced the evaluation results. Longer transcripts were associated with higher clinical educator consensus ratings and larger absolute discrepancies between the clinical educator consensus ratings and both GenAI model scores. However, these exploratory associations do not establish causality. Transcript length may reflect training modality, verbosity, transcript completeness, interaction structure, case-specific characteristics, or the quality of resident performance.

### Comparison With Prior Work

A critical insight emerges when comparing these results to our previous investigation using GPT-4 [43]. In the prior study, GPT-4 exhibited a tendency toward score inflation, assigning significantly higher scores than physicians in domains such as clinical reasoning (median 5.0, IQR 5.0-5.0 vs median 4.0, IQR 3.0-4.0; *P*<.001), and management (median 6.0, IQR 5.0-6.0 vs median 4.0, IQR 2.5-4.5; *P*=.002).

In contrast, this study showed a scoring tendency in the opposite direction, with lower scores assigned by GPT-5.2 Thinking and Gemini 3.0 Pro than by the clinical educator consensus ratings. As the prior and current studies differed in datasets, learner populations, rubrics, prompts, platforms, and experimental conditions, the observed difference in scoring direction cannot be attributed to model evolution alone. For example, in the management domain, where GPT-4 previously overestimated performance, GPT-5.2 Thinking underestimated it (mean 2.93 vs human 5.20). Despite this reversal in scoring direction, the lack of reliability remains consistent between the two studies. Both the previous study (ICC between GPT-4 and clinical educator consensus score for total score=0.00) and the current study (ICC between GPT-5.2 Thinking and clinical educator consensus score for overall score=0.04) show that the evaluated configurations did not rank residents consistently with educator consensus ratings. This scoring direction may reflect differences in model architecture, training, alignment strategies, prompt, rubric interpretation, or other experimental conditions. However, this study cannot determine the mechanism underlying this change. The observed score deflation may indicate that these models applied stricter or more literal rubric interpretation under the tested prompt conditions, but the present study cannot determine the underlying mechanism [63].

### Future Directions

Future research should evaluate larger and more diverse datasets across multiple institutions, languages, and clinical scenarios to assess the robustness and generalizability of GenAI-based evaluation. Incorporating multimodal inputs, such as audio, video, and nonverbal behavior, may improve model alignment with human assessment in communication and professionalism domains [64]. Methodologically, ensemble approaches combining multiple models, calibration techniques, or rubric anchoring using human-labeled training data may reduce systematic bias and improve agreement [65]. Furthermore, future investigations should use mixed methods designs, combining quantitative scoring analysis with qualitative thematic analysis of GenAI-generated feedback.

Future studies should separately evaluate representative educational use cases: summative scoring, formative narrative feedback, calibration support for clinical educators, and learner self-reflection. Evidence supporting one use case should not be assumed to establish validity for the others. As this study collected and analyzed only numerical scores, it cannot determine whether the models’ qualitative feedback would be accurate, educationally useful, or acceptable to learners and educators. Future studies should explicitly instruct models to generate narrative feedback, collect those outputs systematically, and evaluate them using qualitative thematic analysis, educator review, and learner-centered outcomes. Finally, integrating explainability mechanisms that provide transparent rationales for scores may enhance educator trust, facilitate error analysis, and support responsible deployment of GenAI systems in medical education.

### Conclusions

In this preliminary, single-center, transcript-based study of a Japanese-language OSCE dataset, uncalibrated single-run zero-shot evaluations by GPT-5.2 Thinking and Gemini 3.0 Pro assigned lower scores than clinical educator consensus ratings. These findings do not support the use of these models as standalone evaluators under the specific conditions tested for Japanese medical interview training. However, the results do not establish that these models generally lack validity across other settings, languages, prompts, model configurations, or assessment formats. Further multicenter studies, refinements in prompting strategies, and model calibration, particularly through specialized training, are required to harmonize GenAI evaluation with human clinical judgment.

## Supplementary material

10.2196/92016 Multimedia Appendix 1 Details of medical interview training.

10.2196/92016 Multimedia Appendix 2 Summaries of the 2 clinical cases used in medical interview training.

10.2196/92016 Multimedia Appendix 3 Details of evaluation for medical interview training.

10.2196/92016 Multimedia Appendix 4 Median and IQR of evaluation scores by evaluator group and domain.

10.2196/92016 Multimedia Appendix 5 Participant-level sensitivity analysis of overall scores.

10.2196/92016 Multimedia Appendix 6 Exploratory calibration analysis of generative artificial intelligence (GenAI) model scores against clinical educator consensus scores.

10.2196/92016 Multimedia Appendix 7 Additional exploratory analysis of transcript length.

10.2196/92016 Checklist 1 CONSORT-eHEALTH (V 1.6.1).

## Abbreviations

AI: artificial intelligence
GenAI: generative artificial intelligence
GPT: generative pretrained transformer
ICC: intraclass correlation coefficient
LLM: large language model
OSCE: Objective Structured Clinical Examination
PGY: postgraduate year

## References

[1] Stoeckle JD, Billings JA (1987). A history of history-taking: the medical interview. J Gen Intern Med.

[2] Hampton JR, Harrison MJ, Mitchell JR, Prichard JS, Seymour C (1975). Relative contributions of history-taking, physical examination, and laboratory investigation to diagnosis and management of medical outpatients. Br Med J.

[3] Keifenheim KE, Teufel M, Ip J (2015). Teaching history taking to medical students: a systematic review. BMC Med Educ.

[4] Lichstein PR, HW WH, JW H (1990). Clinical Methods: The History, Physical, and Laboratory Examinations.

[5] Eggly S (2002). Physician-patient co-construction of illness narratives in the medical interview. Health Commun.

[6] Akobeng AK (2007). Understanding diagnostic tests 2: likelihood ratios, pre- and post-test probabilities and their use in clinical practice. Acta Paediatr.

[7] Kent P, Hancock MJ (2016). Interpretation of dichotomous outcomes: sensitivity, specificity, likelihood ratios, and pre-test and post-test probability. J Physiother.

[8] Yang D, Fineberg HV, Cosby K (2021). Diagnostic excellence. JAMA.

[9] Watari T, Schiff GD (2023). Diagnostic excellence in primary care. J Gen Fam Med.

[10] Singh H, Connor DM, Dhaliwal G (2022). Five strategies for clinicians to advance diagnostic excellence. BMJ.

[11] Lipkin M, Quill TE, Napodano RJ (1984). The medical interview: a core curriculum for residencies in internal medicine. Ann Intern Med.

[12] Novack DH, Volk G, Drossman DA, Lipkin M (1993). Medical interviewing and interpersonal skills teaching in US medical schools. Progress, problems, and promise. JAMA.

[13] Fava GA, Sonino N, Aron DC (2024). Clinical interviewing: an essential but neglected method of medicine. Psychother Psychosom.

[14] Benbassat J, Baumal R (2009). A proposal for overcoming problems in teaching interviewing skills to medical students. Adv Health Sci Educ Theory Pract.

[15] Bokken L, Rethans JJ, Scherpbier A, van der Vleuten CPM (2008). Strengths and weaknesses of simulated and real patients in the teaching of skills to medical students: a review. Simul Healthcare.

[16] Cleland JA, Abe K, Rethans JJ (2009). The use of simulated patients in medical education: AMEE Guide No 42. Med Teach.

[17] Lucchetti G, Ezequiel OS, Lucchetti ALG (2017). An OSCE with very limited resources: is it possible?. Med Teach.

[18] Bergus GR, Woodhead JC, Kreiter CD (2009). Trained lay observers can reliably assess medical students’ communication skills. Med Educ.

[19] Abe K, Suzuki T, Fujisaki K, Ban N (2007). Demographic characteristics of standardized patients (SPs) and their satisfaction and burdensome in Japan: the first report of a nationwide survey [Article in Japanese]. Igaku Kyoiku.

[20] Maloney S, Haines T (2016). Issues of cost-benefit and cost-effectiveness for simulation in health professions education. Adv Simul (Lond).

[21] Gormley G (2011). Summative OSCEs in undergraduate medical education. Ulster Med J.

[22] Hyde S, Fessey C, Boursicot K, MacKenzie R, McGrath D (2022). OSCE rater cognition - an international multi-centre qualitative study. BMC Med Educ.

[23] Chong L, Taylor S, Haywood M, Adelstein BA, Shulruf B, Huh S (2017). The sights and insights of examiners in objective structured clinical examinations. J Educ Eval Health Prof.

[24] McManus IC, Thompson M, Mollon J (2006). Assessment of examiner leniency and stringency ('hawk-dove effect’) in the MRCP(UK) clinical examination (PACES) using multi-facet Rasch modelling. BMC Med Educ.

[25] Wood TJ (2014). Exploring the role of first impressions in rater-based assessments. Adv Health Sci Educ Theory Pract.

[26] Oliveira Franco RL, Martins Machado JL, Satovschi Grinbaum R, Martiniano Porfírio GJ (2019). Barriers to outpatient education for medical students: a narrative review. Int J Med Educ.

[27] Pereira DSM, Falcão F, Nunes A, Santos N, Costa P, Pêgo JM (2023). Designing and building OSCEBot ® for virtual OSCE - performance evaluation. Med Educ Online.

[28] Bjerring JC, Busch J (2021). Artificial intelligence and patient-centered decision-making. Philos Technol.

[29] Gazquez-Garcia J, Sánchez-Bocanegra CL, Sevillano JL (2025). AI in the health sector: systematic review of key skills for future health professionals. JMIR Med Educ.

[30] Delipetrev B, Tsinaraki C, Kostic U (2020). Historical evolution of artificial intelligence. https://publications.jrc.ec.europa.eu/repository/handle/JRC120469.

[31] Chen L, Chen P, Lin Z (2020). Artificial intelligence in education: a review. IEEE Access.

[32] Lucas HC, Upperman JS, Robinson JR (2024). A systematic review of large language models and their implications in medical education. Med Educ.

[33] Sai S, Gaur A, Sai R, Chamola V, Guizani M, Rodrigues J (2024). Generative AI for transformative healthcare: a comprehensive study of emerging models, applications, case studies, and limitations. IEEE Access.

[34] Chang Y, Wang X, Wang J (2024). A survey on evaluation of large language models. ACM Trans Intell Syst Technol.

[35] Holderried F, Stegemann-Philipps C, Herschbach L (2024). A generative pretrained transformer (GPT)–powered chatbot as a simulated patient to practice history taking: prospective, mixed methods study. JMIR Med Educ.

[36] Potter L, Jefferies C (2024). Enhancing communication and clinical reasoning in medical education: building virtual patients with generative AI. Future Healthcare J.

[37] Achiam J, Adler S, OpenAI (2024). GPT-4 technical report. arXiv.

[38] Team G, Anil R, Borgeaud S, Wu Y, Alayrac JB, Yu J (2025). Gemini: a family of highly capable multimodal models. arXiv.

[39] Saab K, Tu T, Weng WH, Tanno R, Stutz D, Wulczyn E (2024). Capabilities of gemini models in medicine. arXiv.

[40] Bridges JM (2024). Computerized diagnostic decision support systems - a comparative performance study of Isabel Pro vs. ChatGPT4. Diagnosis (Berl).

[41] Restrepo D, Rodman A, Abdulnour RE (2024). Conversations on reasoning: large language models in diagnosis. J Hosp Med.

[42] Kung TH, Cheatham M, Medenilla A (2023). Performance of ChatGPT on USMLE: potential for AI-assisted medical education using large language models. PLOS Digit Health.

[43] Yokose M, Hirosawa T, Sakamoto T (2025). The validity of generative artificial intelligence in evaluating medical students in objective structured clinical examination: experimental study. JMIR Form Res.

[44] Gu SS, Iwasawa Y, Kojima T, Matsuo Y, Reid M Large language models are zero-shot reasoners.

[45] Yu F, Zhang H, Tiwari P, Wang B (2024). Natural language reasoning, a survey. ACM Comput Surv.

[46] Schouten BC, Meeuwesen L (2006). Cultural differences in medical communication: a review of the literature. Patient Educ Couns.

[47] Hydén LC, Mishler EG (1999). Language and medicine. Ann Rev Appl Linguist.

[48] Yamamoto A, Koda M, Ogawa H (2024). Enhancing medical interview skills through AI-simulated patient interactions: nonrandomized controlled trial. JMIR Med Educ.

[49] Hirosawa T, Yokose M, Sakamoto T (2025). Utility of generative artificial intelligence for Japanese medical interview training: randomized crossover pilot study. JMIR Med Educ.

[50] (2023). Introducing GPTs. OpenAI.

[51] Harden RM, Gleeson FA (1979). Assessment of clinical competence using an objective structured clinical examination (OSCE). Med Educ.

[52] Madrazo L, Lee CB, McConnell M, Khamisa K (2018). Self-assessment differences between genders in a low-stakes objective structured clinical examination (OSCE). BMC Res Notes.

[53] Koo TK, Li MY (2016). A guideline of selecting and reporting intraclass correlation coefficients for reliability research. J Chiropr Med.

[54] Armstrong RA (2014). When to use the Bonferroni correction. Ophthalmic Physiol Opt.

[55] Palatucci M, Pomerleau D, Hinton GE, Mitchell TM (2009). Zero-shot learning with semantic output codes. Adv Neural Inf Process Syst.

[56] Guo D, Yang D, Zhang H, Song J, Zhang R, Xu R (2026). DeepSeek-R1: incentivizing reasoning capability in LLMs via reinforcement learning. arXiv.

[57] (2025). Introducing GPT-5.2. OpenAI.

[58] (2026). Introducing ChatGPT Health. OpenAI.

[59] Vinyals O, Blundell C, Lillicrap T, Wierstra D Matching networks for one shot learning. https://proceedings.neurips.cc/paper_files/paper/2016/file/90e1357833654983612fb05e3ec9148c-Paper.pdf.

[60] Brown T, Mann B, Ryder N, Subbiah M, Kaplan JD, Dhariwal P (2020). Language models are few-shot learners. Adv Neural Inf Process Syst.

[61] Liévin V, Hother CE, Motzfeldt AG, Winther O (2024). Can large language models reason about medical questions?. Patterns (N Y).

[62] Haviari S, de Tymowski C, Burnichon N (2024). Measuring and correcting staff variability in large-scale OSCEs. BMC Med Educ.

[63] Sorin V, Brin D, Barash Y (2024). Large language models and empathy: systematic review. J Med Internet Res.

[64] Acosta JN, Falcone GJ, Rajpurkar P, Topol EJ (2022). Multimodal biomedical AI. Nat Med.

[65] Ganaie MA, Hu M, Malik AK, Tanveer M, Suganthan PN (2022). Ensemble deep learning: a review. Eng Appl Artif Intell.

