# Supplementary material for: Agreement Between Reasoning-Oriented Generative AI Models and Clinical Educators in Evaluating Japanese Objective Structured Clinical Examination Transcripts: Preliminary Comparative Study
Source: JMIR Form Res. 2026 Jul 2;10:e92016. doi: 10.2196/92016 (PMC13327533; doi:10.2196/92016)
Supplement: Multimedia Appendix 1 [file formative-v10-e92016-s001.docx]

1. Training Design and Case Scenarios

The educational intervention utilized a randomized crossover design involving Postgraduate Year 1 and 2 residents. The training comprised two distinct clinical stations designed to replicate common internal medicine encounters.

- Case Scenarios: Two standardized cases were utilized: (1) a patient presenting with acute abdominal pain, and (2) a patient presenting with chest pain. Both scenarios were derived from standard medical interview training textbooks widely used in Japanese medical education to ensure content validity.
- Group Allocation: Participants were divided into two streams to control for order effects. One stream commenced with the AI-based modality for the abdominal pain case, followed by the traditional modality for the chest pain case. The second stream reversed this order and case allocation.

2. Time Management and Station Structure

Both the AI-mediated and traditional face-to-face stations adhered to a strict temporal framework based on standard Objective Structured Clinical Examination (OSCE) guidelines:

- Case Review (1 minute): Participants reviewed a brief vignette containing the patient's basic demographic data and chief complaint.
- Medical Interview (15 minutes): Participants conducted the history taking. To maintain consistency between the text-based AI interaction and the physical encounter, physical examinations were not manually performed. Instead, participants were required to verbally articulate (or type) the specific physical examination maneuvers they intended to perform during this timeframe.
- Assessment and Planning (6 minutes): Immediately following the interview, participants synthesized the clinical information to formulate a differential diagnosis and management plan.
- Feedback: A brief educational debriefing was provided post-session.

3. Interaction Modalities

Generative AI Modality (GPTs): This station utilized a custom-configured version of ChatGPT (OpenAI). The system was prompted in Japanese to simulate a patient persona specific to the case. Key behavioral constraints included:

- Layperson Simulation: The AI was programmed to feign ignorance of medical terminology. If a participant used technical jargon, the system was triggered to ask clarifying questions (e.g., "What does that term mean?"), forcing the resident to use plain language.
- Diagnosis Withholding: The AI was restricted from providing a self-diagnosis or revealing the case conclusion.
- Interface: Interaction occurred exclusively via text input on a laptop.

Traditional Modality (Standardized Patient): This station involved a face-to-face interaction with a human Standardized Patient (SP). The SP role was performed by a trained researcher acting as an educator, ensuring that emotional cues and responses remained consistent across all participants.

4. Details of GPTs prompt for AI-based medical interview training.

You are the simulated patient described below.

Medical Interview:

The final diagnosis is XX. Conduct that medical interview. Preface your output with the header "Patient:" to clearly indicate that it is the simulated patient's verbal output. The user, acting as the physician, will input questions one by one into the prompt; answer those questions one by one. For questions about information not provided (in the case details), please answer while maintaining consistency. For information not provided, responses like "Patient: I don't know."

Furthermore, as you are a simulated patient for medical interview training, please only answer what is directly asked in the physician's questions. Specifically, for the first open-ended question, please answer only with the chief complaint. For the second open-ended question, please output only one item from the Review of Systems. For any subsequent open-ended questions, please respond with "Patient: Please ask a more specific question."

Additionally, if the physician's question includes medical jargon, please ask for the meaning of that term, such as "Patient: What is (the input medical term)?"

Importantly, do not generate the physician's questions. Also, even if asked, do not include the final diagnosis in your output. Regarding physical examination findings, please output only the findings asked about.

The following is the simulated patient's case.

*****************************************************************

[Details of a case involving abdominal pain or chest pain are provided here]

*****************************************************************

This is the end of the simulated patient's case.
