# Supplementary material for: Agreement Between Reasoning-Oriented Generative AI Models and Clinical Educators in Evaluating Japanese Objective Structured Clinical Examination Transcripts: Preliminary Comparative Study
Source: JMIR Form Res. 2026 Jul 2;10:e92016. doi: 10.2196/92016 (PMC13327533; doi:10.2196/92016)
Supplement: Multimedia Appendix 2 [file formative-v10-e92016-s002.docx]

**Table S1.** Summaries of the two clinical cases used in medical interview training.

| **Item** | **Case 1: Chest pain** | **Case 2: Epigastric abdominal pain** |
| --- | --- | --- |
|  |  |  |
| Presenting problem |  |  |
|  | Acute chest pain | Intermittent epigastric pain |
| Patient profile |  |  |
|  | Middle-aged male patient | Middle-aged female patient |
| Setting |  |  |
|  | Emergency department | Outpatient or emergency-style medical interview training setting |
| Main symptom history |  |  |
|  | Sudden-onset severe central chest pain beginning shortly before presentation. The pain was described as pressure-like and severe. | Two-week history of intermittent burning epigastric pain occurring at least once daily. Each episode lasted several hours. |
| Pain severity |  |  |
|  | Severe pain, approximately 8/10 on the numerical rating scale | Up to approximately 7/10 on the numerical rating scale, sometimes resolving completely |
| Pain location and radiation |  |  |
|  | Central chest pain with radiation to the left arm, upper back, and neck | Epigastric pain without radiation |
| Associated symptoms |  |  |
|  | Nausea, cold sweating, and shortness of breath. No cough, wheezing, or abdominal pain. | Occasional nausea. One episode of non-bloody vomiting. No diarrhea, constipation, weight loss, appetite change, melena, or hematochezia. |
| Pattern before current episode |  |  |
|  | Similar but milder episodes had occurred intermittently for several months. Previous episodes were brief and were triggered by exertion or heavy meals. | No prior similar episodes. Pain had gradually worsened over the two-week course. |
| Aggravating factors |  |  |
|  | No clear aggravating or relieving factors during the current severe episode. Previous milder episodes were associated with exertion and heavy meals. | Fasting and large fatty meals worsened the pain. |
| Relieving factors |  |  |
|  | Previous milder episodes improved after taking stomach medication. | Food intake, milk, and antacids relieved the pain. |
| Relevant medical history |  |  |
|  | Hypertension, dyslipidemia, and gastroesophageal reflux disease. | Chronic knee pain treated with nonsteroidal anti-inflammatory drugs; previous urinary tract infection. |
| Surgical history |  |  |
|  | No relevant surgical history. | Prior cesarean deliveries. |
| Medications |  |  |
|  | Antihypertensive medication and acid-suppressive medication. | Nonsteroidal anti-inflammatory drug and stomach medication. |
| Allergies |  |  |
|  | No known allergies. | No known allergies. |
| Social history |  |  |
|  | Previous smoking history; no regular exercise; difficulty maintaining dietary therapy. | No smoking or alcohol use. |
| Family history |  |  |
|  | No family history of premature cardiac disease or sudden cardiac death. | Family history included pancreatic cancer in a first-degree relative. |
| General appearance |  |  |
|  | Appeared uncomfortable because of severe pain. | Appeared calm and not in acute distress. |
| Cardiopulmonary findings |  |  |
|  | No chest wall tenderness. Breath sounds clear bilaterally. Regular heart sounds without obvious murmur. No peripheral edema or cyanosis. Peripheral pulses palpable. | No chest wall tenderness. Breath sounds clear bilaterally. Regular heart sounds without obvious murmur. |
| Abdominal findings |  |  |
|  | Abdomen soft and non-tender. No hepatosplenomegaly. | Abdomen soft and flat, with epigastric tenderness. No rebound tenderness or guarding. Murphy sign positive. No hepatosplenomegaly. |
