# Supplementary material for: Agreement Between Reasoning-Oriented Generative AI Models and Clinical Educators in Evaluating Japanese Objective Structured Clinical Examination Transcripts: Preliminary Comparative Study
Source: JMIR Form Res. 2026 Jul 2;10:e92016. doi: 10.2196/92016 (PMC13327533; doi:10.2196/92016)
Supplement: Multimedia Appendix 3 [file formative-v10-e92016-s003.docx]

1. Data Processing and Blinding

To ensure objective assessment and eliminate bias regarding the interaction modality, all interview data were converted into a uniform text format prior to evaluation.

- Transcription: Traditional face-to-face interviews were video-recorded and manually transcribed. AI-based sessions were exported as text logs.
- Anonymization: Identifiers specific to the modality were stripped from the text. For instance, system-generated headers such as "ChatGPT" were replaced with "Patient," and the introductory phase (self-introduction) was removed.
- Randomization: The resulting text files were randomized, ensuring evaluators were blinded to both the participant's identity and whether the interview was conducted with a human or an AI.

2. Scoring Metrics

Two independent board-certified physicians evaluated the transcripts. Discrepancies in scoring were resolved through a consensus discussion. Performance was quantified using a six-point Likert scale (1 = Inferior/Very Poor; 6 = Excellent).

3. Assessment Domains

The evaluation rubric was adapted from standard OSCE criteria and comprised six distinct domains:

1. Patient Care and Communication Skills: Assessed the participant's empathy, politeness, and ability to build rapport using appropriate language.
2. Thoroughness of History-Taking: Evaluated the comprehensive gathering of the history of present illness (HPI), past medical history, and social history relevant to the complaint.
3. Physical Examination Proficiency: Scored based on the appropriateness and completeness of the physical examination maneuvers the participant verbally proposed or typed during the session.
4. Accuracy and Clarity of Transcription: Evaluated the logical flow and clarity of the conversation record (Note: This domain assessed the communication clarity within the transcript, not the formatting of a medical chart). While standard OSCE rubrics typically evaluate "Medical Recording" (e.g., the quality of written SOAP notes), this study design did not require participants to generate formal medical records. Consequently, this domain was modified to evaluate the logical flow, coherence, and clarity of the interview dialogue and the participant's subsequent case presentation, rather than the formatting of a traditional medical chart.
5. Clinical Reasoning Capability: Assessed the logical derivation of differential diagnoses based on the information gathered.
6. Overall Patient Management Strategies: Evaluated the appropriateness of the proposed diagnostic tests, treatments, and follow-up plans.
