# Supplementary material for: Agreement Between Reasoning-Oriented Generative AI Models and Clinical Educators in Evaluating Japanese Objective Structured Clinical Examination Transcripts: Preliminary Comparative Study
Source: JMIR Form Res. 2026 Jul 2;10:e92016. doi: 10.2196/92016 (PMC13327533; doi:10.2196/92016)
Supplement: Multimedia Appendix 4 [file formative-v10-e92016-s004.docx]

**Table S1.** Median and interquartile range of evaluation scores by evaluator group and domain.

|  | Median scores with a 6-point Likert scale (interquartile range) | | |
| --- | --- | --- | --- |
|  | GPT-5.2 Thinking | Gemini 3.0 Pro | Clinical educator consensus score |
|  |  |  |  |
| **Overall** |  |  |  |
|  | 4 (3-4) | 4 (3-5) | 5 (5-6) |
| **Patient care and communication** |  |  |  |
|  | 4 (4-4) | 5 (4-5) | 5 (5-5) |
| **History taking** |  |  |  |
|  | 4 (4-4) | 4.5 (4-5) | 5 (5-5.25) |
| **Physical examination** |  |  |  |
|  | 4 (4-4) | 4 (3-5) | 6 (5-6) |
| **Accuracy and organization of clinical information** |  |  |  |
|  | 4 (4-4) | 4 (3-5) | 5 (5-5.25) |
| **Clinical reasoning** |  |  |  |
|  | 3 (3-4) | 4 (3-5) | 5 (4.75-6) |
| **Management** |  |  |  |
|  | 3 (3-3) | 4 (3-4) | 5 (5-6) |
