# Supplementary material for: Agreement Between Reasoning-Oriented Generative AI Models and Clinical Educators in Evaluating Japanese Objective Structured Clinical Examination Transcripts: Preliminary Comparative Study
Source: JMIR Form Res. 2026 Jul 2;10:e92016. doi: 10.2196/92016 (PMC13327533; doi:10.2196/92016)
Supplement: Multimedia Appendix 5 [file formative-v10-e92016-s005.docx]

**Table S1.** Participant-level sensitivity analysis of overall scores.

|  | Mean scores with a 6-point Likert scale (95% CI) | | | *P* value^a^ | | |
| --- | --- | --- | --- | --- | --- | --- |
|  | GPT-5.2 Thinking | Gemini 3.0 Pro | Clinical educator consensus score | GPT-5.2 Thinking vs Clinical educator consensus score | Gemini 3.0 Pro vs Clinical educator consensus score | GPT-5.2 Thinking vs Gemini 3.0 Pro |
|  |  |  |  |  |  |  |
| **Overall** |  |  |  |  |  |  |
|  | 3.68 (3.59-3.76) | 4.09 (3.91-4.28) | 5.18 (4.99-5.37) | *P*<.001 | *P*<.001 | *P*<.001 |

^a^Statistical comparisons were conducted using the Wilcoxon signed-rank test.

| **Comparison** | **Difference definition** | **Mean difference** | **95% CI** | **n persons** |
| --- | --- | --- | --- | --- |
| Clinical educator consensus score vs GPT-5.2 Thinking | GPT-5.2 Thinking - Clinical educator consensus score |  |  |  |
|  |  | -1.50 | -1.68 to -1.32 | 20 |
| Clinical educator consensus score vs Gemini 3.0 Pro | Gemini 3.0 Pro - Clinical educator consensus score |  |  |  |
|  |  | -1.09 | -1.26 to -0.91 | 20 |
| GPT-5.2 Thinking vs Gemini 3.0 Pro | Gemini 3.0 Pro - GPT-5.2 Thinking |  |  |  |
|  |  | 0.42 | 0.25 to 0.58 | 20 |
