# Supplementary material for: Agreement Between Reasoning-Oriented Generative AI Models and Clinical Educators in Evaluating Japanese Objective Structured Clinical Examination Transcripts: Preliminary Comparative Study
Source: JMIR Form Res. 2026 Jul 2;10:e92016. doi: 10.2196/92016 (PMC13327533; doi:10.2196/92016)
Supplement: Multimedia Appendix 6 [file formative-v10-e92016-s006.docx]

**Table S1.** Exploratory calibration analysis of GAI model scores against clinical educator consensus scores.

| **Domain** | **Model** | **Mean signed difference, 95% CI; LoA** | **Mean absolute difference** | **Within 1 point, %** | **Spearman ρ** | **Ordering concordance / model tie rate** | **Linear regression slope, R²** |
| --- | --- | --- | --- | --- | --- | --- | --- |
|  |  |  |  |  |  |  |  |
| Overall | GPT-5.2 Thinking |  |  |  |  |  |  |
|  |  | -1.50, -1.68 to -1.33; -2.59 to -0.42 | 1.50 | 17.5% | 0.31 | 48.9% / 23.8% | 0.15, 0.12 |
| Overall | Gemini 3.0 Pro |  |  |  |  |  |  |
|  |  | -1.09, -1.26 to -0.91; -2.15 to -0.02 | 1.09 | 60.0% | 0.62 | 69.2% / 6.6% | 0.60, 0.34 |
| A | GPT-5.2 Thinking |  |  |  |  |  |  |
|  |  | -1.18, -1.42 to -0.93; -2.71 to 0.36 | 1.23 | 62.5% | 0.16 | 10.0% / 87.0% | 0.05, 0.02 |
| A | Gemini 3.0 Pro |  |  |  |  |  |  |
|  |  | -0.82, -1.10 to -0.55; -2.54 to 0.89 | 1.03 | 80.0% | 0.49 | 56.6% / 30.5% | 0.41, 0.16 |
| B | GPT-5.2 Thinking |  |  |  |  |  |  |
|  |  | -1.27, -1.49 to -1.06; -2.61 to 0.06 | 1.27 | 60.0% | 0.26 | 27.0% / 65.3% | 0.17, 0.06 |
| B | Gemini 3.0 Pro |  |  |  |  |  |  |
|  |  | -0.72, -0.97 to -0.48; -2.20 to 0.75 | 0.78 | 85.0% | 0.32 | 46.9% / 37.2% | 0.42, 0.14 |
| C | GPT-5.2 Thinking |  |  |  |  |  |  |
|  |  | -1.55, -1.80 to -1.30; -3.08 to -0.02 | 1.55 | 42.5% | 0.11 | 26.4% / 55.5% | 0.14, 0.04 |
| C | Gemini 3.0 Pro |  |  |  |  |  |  |
|  |  | -1.38, -1.71 to -1.04; -3.44 to 0.69 | 1.38 | 55.0% | 0.44 | 62.3% / 20.5% | 0.74, 0.20 |
| D | GPT-5.2 Thinking |  |  |  |  |  |  |
|  |  | -1.23, -1.45 to -1.00; -2.59 to 0.14 | 1.23 | 62.5% | 0.28 | 23.8% / 70.9% | 0.17, 0.10 |
| D | Gemini 3.0 Pro |  |  |  |  |  |  |
|  |  | -1.00, -1.25 to -0.75; -2.54 to 0.54 | 1.00 | 75.0% | 0.52 | 63.4% / 25.5% | 0.75, 0.33 |
| E | GPT-5.2 Thinking |  |  |  |  |  |  |
|  |  | -1.52, -1.85 to -1.20; -3.51 to 0.46 | 1.58 | 47.5% | 0.24 | 39.2% / 41.9% | 0.20, 0.08 |
| E | Gemini 3.0 Pro |  |  |  |  |  |  |
|  |  | -0.98, -1.42 to -0.53; -3.69 to 1.74 | 1.33 | 62.5% | 0.14 | 41.3% / 29.3% | 0.16, 0.02 |
| F | GPT-5.2 Thinking |  |  |  |  |  |  |
|  |  | -2.28, -2.56 to -1.99; -3.99 to -0.56 | 2.28 | 12.5% | 0.23 | 24.1% / 66.9% | 0.14, 0.10 |
| F | Gemini 3.0 Pro |  |  |  |  |  |  |
|  |  | -1.62, -1.92 to -1.33; -3.44 to 0.19 | 1.62 | 50.0% | 0.42 | 57.8% / 25.5% | 0.51, 0.25 |

Domain labels: A, patient care and communication; B, history taking; C, physical examination; D, accuracy and organization of clinical information; E, clinical reasoning; F, management. Abbreviations: CI, confidence interval; LoA, limits of agreement; GAI, generative artificial intelligence.

**Methods**

As a post hoc exploratory analysis, we further characterized agreement between each GAI model and the clinical educator consensus ratings. Analyses were conducted at the transcript level using all 40 transcripts. The overall score was defined as the mean score across the six rubric domains.

For each transcript, the signed difference was calculated as the GAI model score minus the clinical educator consensus score. Negative values therefore indicate that the GAI model assigned a lower score than the clinical educator consensus rating. We calculated the mean signed difference with its 95% confidence interval, the 95% limits of agreement, the mean absolute difference, and the proportion of GAI model scores that were within 1 point of the clinical educator consensus score.

We also calculated Spearman rank correlation coefficients to assess associations between the GAI model scores and the clinical educator consensus ratings. To explore preservation of the relative ordering of resident performance, we calculated pairwise ordering concordance and the model tie rate. Finally, we fitted exploratory linear regression models with the GAI model score as the dependent variable and the clinical educator consensus score as the independent variable. Linear regression slopes and coefficients of determination (R²) are reported descriptively.

These analyses were exploratory and were not intended to establish the validity of a recalibrated scoring model. Prospective calibration would require predefined educator-approved rubric anchors, larger datasets, and independent validation samples.

**Summary**

Both GAI models showed systematic negative bias compared with clinical educator consensus scores, indicating systematically lower score assignment. The degree of score deflation was larger for GPT-5.2 Thinking than for Gemini 3.0 Pro and was greatest in the management domain. For the overall score, the mean bias was -1.50 points for GPT-5.2 Thinking and -1.09 points for Gemini 3.0 Pro.

However, the disagreement was not explained only by a simple additive score offset. GPT-5.2 Thinking showed weak rank correlation with clinical educator scores and frequent tied scores, suggesting limited discrimination of relative resident performance under the tested conditions. Gemini 3.0 Pro showed better rank correlation and ordering preservation, particularly for the overall score, indicating that prospective calibration may warrant further evaluation. Nevertheless, ordering preservation remained imperfect, and these exploratory findings do not establish that calibrated GAI scores are suitable for standalone summative assessment. Future studies should prospectively evaluate calibration using larger datasets and independent validation samples.

**Retrospective Illustrative Examples of Scoring Differences**

To provide additional context for the observed differences between the GAI model scores and the clinical educator consensus ratings, we retrospectively reviewed three anonymized transcripts representing selected scoring patterns. These examples were selected for illustration after completion of the primary analysis. They were not provided to either GAI model during the original evaluations and should not be interpreted as a formal qualitative error analysis or as prospectively defined rubric anchors.

**Interpretation**

These illustrative examples suggest that the score discrepancies may partly reflect differences in the level of explicit documentation required by the evaluators. In some transcripts, the clinical educators judged that the resident had adequately addressed life-threatening conditions and demonstrated performance appropriate for supervised practice, even when the summarized assessment and plan were concise. In contrast, the GAI models may have required more explicit detail regarding the differential diagnosis, prioritization, and management strategy to assign higher scores.

However, the present analysis cannot determine whether the GAI models applied an overly strict standard, whether the clinical educators applied a more pragmatic supervised-practice standard, or whether both factors contributed. Prospective studies should test educator-approved rubric anchors and few-shot calibration examples using independent validation datasets.

**Table S2.** Retrospective illustrative examples of differences between GAI model scores and clinical educator consensus ratings.

| Example number | Clinical educator consensus A–F | GPT-5.2 Thinking A–F | Gemini Pro 3.0 A–F | Evaluation  characteristic | Retrospective descriptive interpretation |
| --- | --- | --- | --- | --- | --- |
| 1 |  |  |  |  |  |
|  | 6, 6, 6, 6, 4, 6 | 4, 4, 3, 4, 3, 2 | 5, 5, 4, 5, 3, 2 | high management-score discrepancy | The summarized assessment and plan were concise. The clinical educators judged that the participant had appropriately considered and planned an evaluation for potentially life-threatening conditions and therefore assigned a high management score. Both GAI models assigned lower scores in clinical reasoning and management. One possible explanation is that the models required more explicit documentation of the differential diagnosis, prioritization, or management plan |
| 2 |  |  |  |  |  |
|  | 6, 6, 6, 6, 6, 6 | 4, 4, 4, 4, 3, 3 | 5, 5, 6, 5, 5, 4 | high educator ratings with lower GAI ratings | The summarized assessment and plan were concise, but the clinical educators judged that the participant had adequately addressed life-threatening conditions and demonstrated performance appropriate for supervised clinical practice. GPT-5.2 Thinking, and to a lesser extent Gemini 3.0 Pro, assigned lower clinical-reasoning and management scores. This pattern may reflect a greater emphasis by the models on explicit detail in the transcript. |
| 3 |  |  |  |  |  |
|  | 4, 4, 5, 4, 3, 3 | 4, 4, 4, 4, 4, 3 | 4, 4, 5, 3, 4, 2 | relatively aligned lower scores | The medical interview and summarized assessment and plan were relatively brief. Both the clinical educators and the GAI models assigned comparatively lower scores. This example indicates that the GAI models were not uniformly more critical in every transcript and that lower scores may partly reflect limited explicit documentation of clinical reasoning and management. |

Note: Domain labels: A, patient care and communication; B, history taking; C, physical examination; D, accuracy and organization of clinical information; E, clinical reasoning; F, management. These examples were retrospectively selected to illustrate scoring patterns. They do not establish the mechanisms underlying disagreement between GAI models and clinical educator consensus ratings. Because model rationales were not collected, the proposed explanations remain hypotheses.
