# Supplementary material for: Agreement Between Reasoning-Oriented Generative AI Models and Clinical Educators in Evaluating Japanese Objective Structured Clinical Examination Transcripts: Preliminary Comparative Study
Source: JMIR Form Res. 2026 Jul 2;10:e92016. doi: 10.2196/92016 (PMC13327533; doi:10.2196/92016)
Supplement: Multimedia Appendix 7 [file formative-v10-e92016-s007.docx]

**Methods for additional exploratory analysis**

Transcript length was measured as the total number of Japanese characters in each original Japanese-language transcript. The overall score for each evaluator group was defined as the mean score across the six rubric domains: patient care and communication skills, history taking, physical examination, accuracy and organization of clinical information, clinical reasoning, and management.

As an additional exploratory analysis, we examined whether transcript length was associated with the overall score assigned by each evaluator group: the clinical educator consensus rating, GPT-5.2 Thinking, and Gemini 3.0 Pro. We also examined whether transcript length was associated with the absolute score discrepancy between each GAI model and the clinical educator consensus rating. The absolute score discrepancy was defined as the absolute difference between the GAI model’s overall score and the clinical educator consensus overall score for each transcript.

Associations between transcript length and evaluation outcomes were assessed using Spearman rank correlation coefficients. Because each resident contributed two transcripts, we additionally conducted a participant-level sensitivity analysis. For each resident, transcript length and evaluation outcomes were averaged across the two stations, yielding one person-level value per resident. Spearman rank correlation analyses were then repeated at the participant level.

To explore possible modality-related differences in transcript verbosity, transcript length was also compared between the traditional and chatbot-based training styles. Because each resident completed one transcript in each training style, this comparison was performed using the paired Wilcoxon signed-rank test.

These analyses were exploratory. Exact P values are reported without adjustment for multiple comparisons and should be interpreted cautiously.

**Results for additional exploratory analysis**

A total of 40 transcripts from 20 residents were analyzed. The Japanese character count ranged from 872 to 5167 characters, with a mean of 1769.1 characters and a median of 1442.5 characters (IQR 1248.5–1818.5).

At the transcript level, Japanese character count was positively correlated with the clinical educator consensus overall score (Spearman ρ=0.55, *P*<.001). In contrast, transcript length was not correlated with the overall score assigned by GPT-5.2 Thinking (ρ=0.00, *P*=.979) or Gemini 3.0 Pro (ρ=0.18, *P*=.263).

Longer transcripts were associated with larger absolute discrepancies between the clinical educator consensus score and the scores assigned by GPT-5.2 Thinking (ρ=0.57, *P*<.001) and Gemini 3.0 Pro (ρ=0.45, *P*=.004).

The participant-level sensitivity analysis showed a broadly similar pattern, although statistical precision was reduced because the analysis included 20 residents. Mean transcript length was positively correlated with the absolute GPT-5.2 Thinking–clinical educator consensus discrepancy (ρ=0.56, *P*=.010). The association between mean transcript length and the absolute Gemini 3.0 Pro–clinical educator consensus discrepancy was similar in magnitude but did not reach the conventional significance threshold (ρ=0.44, *P*=.051). Mean transcript length was positively correlated with the clinical educator consensus score, although the association did not reach the conventional significance threshold at the participant level (ρ=0.44, *P*=.055). Participant-level correlations between transcript length and the GAI model scores were not statistically significant.

Transcript length also differed according to training modality. Traditional-style transcripts were longer than chatbot-based transcripts. The median Japanese character count was 1754.5 (IQR 1395.25–2558.25; range 1010–5167) for the traditional style and 1280.5 (IQR 1141–1527; range 872–2343) for the chatbot-based style. The difference was statistically significant using the paired Wilcoxon signed-rank test (*P*=.001).

These exploratory findings do not establish that transcript length directly caused the observed scoring discrepancies. Transcript length may reflect several related factors, including training modality, verbosity, transcript completeness, interaction structure, case-specific characteristics, or the quality of the resident’s performance. Larger studies using mixed-effects models are needed to account simultaneously for resident-level clustering, clinical case effects, and training-modality differences.

**Table S1.** Associations between Japanese character count and evaluation outcomes.

| Evaluation outcome | Transcript-level analysis, n=40: Spearman ρ | *P* value | Participant-level sensitivity analysis, n=20: Spearman ρ | *P* value |
| --- | --- | --- | --- | --- |
| Clinical educator consensus overall score |  |  |  |  |
|  | 0.55 | <.001 | 0.44 | .055 |
| GPT-5.2 Thinking overall score |  |  |  |  |
|  | 0.00 | .979 | −0.26 | .260 |
| Gemini 3.0 Pro overall score |  |  |  |  |
|  | 0.18 | .263 | 0.06 | .805 |
| Absolute GPT-5.2 Thinking–clinical educator consensus discrepancy |  |  |  |  |
|  | 0.57 | <.001 | 0.56 | .010 |
| Absolute Gemini 3.0 Pro–clinical educator consensus discrepancy |  |  |  |  |
|  | 0.45 | .004 | 0.44 | .051 |

Notes: The overall score was defined as the mean score across the six rubric domains. The absolute score discrepancy was defined as the absolute difference between each GAI model’s overall score and the clinical educator consensus overall score. For the participant-level sensitivity analysis, transcript length and evaluation outcomes were averaged across the two stations completed by each resident. These analyses were exploratory, and P values were not adjusted for multiple comparisons.

Supplemental Table 7-2. Japanese character count according to training modality.

| Training modality | Number of transcripts | Mean Japanese character count | Median (IQR) | Range |
| --- | --- | --- | --- | --- |
| Traditional style |  |  |  |  |
|  | 20 | 2150.85 | 1754.5 (1395.3–2558.3) | 1010–5167 |
| Chatbot-based style |  |  |  |  |
|  | 20 | 1387.25 | 1280.5 (1141–1527) | 872–2343 |

Note: Transcript length was compared between training modalities using the paired Wilcoxon signed-rank test because each resident completed one transcript in each training style. Traditional-style transcripts were significantly longer than chatbot-based transcripts (*P*=.001).
